# Prevalence of Unclassified Bacteria in Tropical Coastal Waters of Malaysia Revealed by Metagenomic Approach

**DOI:** 10.1128/genomeA.00419-14

**Published:** 2014-05-08

**Authors:** Kok-Gan Chan, Teik-Min Chong

**Affiliations:** Division of Genetics and Molecular Biology, Institute of Biological Sciences, Faculty of Science, University of Malaya, Kuala Lumpur, Malaysia

## Abstract

The metagenomes of marine prokaryotes from coastal seawaters in Malaysia are reported in this study. The investigation of the microbial communities using 16S rRNA gene amplicon metagenomic sequencing revealed that majority of the bacteria in the seawater samples remain unclassified.

## GENOME ANNOUNCEMENT

Microbes are inevitably of great significance in terms of mediating major biogeochemical cycles and enabling the fundamental ecosystem functioning in the ocean (1). As anthropogenic activities have been described to promote changes in oceanic pH, ultraviolet radiation, temperature, and salinity, it would be crucial to further understand the patterns in microbial distributions in response to the changes in the environment (2, 3). This study entails the composition of marine prokaryotes from an urbanized estuary and a fishing village in the coastline of Selangor, Malaysia. The composition and structure of microbial communities inhabiting the seawater were investigated using 16S rRNA gene amplicon metagenomic sequencing.

Seawater at the surface of the littoral zone was collected from an estuary in Sabak Bernam, Selangor, Malaysia (SB), and a fishing village in Sekinchan, Selangor, Malaysia (BSM). The GPS coordinates for the sampling sites are N03°41.379′ E100°56.010′ and N03°30.079′ E101°05.689′, respectively. Immediately after sampling, 1 liter of the seawater from each sampling site was filtered (pore size of 0.22 µm; Sartorius, Germany) followed by DNA extraction using a modified cethyltrimethylammonium bromide DNA extraction protocol (4, 5). PCR amplification of 16S rRNA genes was then performed using primers flanking the V3 to V6 regions (6). Sequencing of the amplified region was accomplished using the Roche/454 GS-FLX Titanium platform. The generated reads were then subjected to quality trimming using CLC Genomics Workbench 5.1 (CLC bio, Denmark) by discarding reads with low quality (<Q20), ambiguous nucleotides, and sequence length <200 nucleotides. Subsequently, annotation and classification were carried out using the MG-RAST server (7) by employing the Ribosomal Database Project (RDP) (8) as the annotation source.

The sequencing yielded a total of 422,255 reads for sample SB and 765,221 reads for sample BSM, with average read lengths of approximately 390 bp and 383 bp, respectively. After quality trimming and analysis in the MG-RAST server, a total of 24 known phyla from *Bacteria* domain were identified in sample SB, whereas 25 known bacterial phyla were detected in sample BSM. On the other hand, a total of 3 known archaeal phyla were detected in both samples comprising members of *Euryarchaeota*, *Crenarchaeota*, and *Thaumarchaeota*.

Interestingly, the most abundant bacterial phylum as revealed by the RDP database was unclassified bacteria, comprising 43.36% (SB) and 43.81% (BSM) of the total *Eubacteria* present. This is followed by the second most abundant bacterial phylum, *Proteobacteria*, and the third, *Firmicutes*, for both samples. Further analysis using the Greengenes (9) and M5RNA databases also verified the presence of unclassified bacteria as the majority of the *Eubacteria* in both samples, even at subsequent taxonomic levels.

The richness in diversity of prokaryotic microbiota from the two coastal waters samples is reported in this study. Interestingly, unusual abundances of unclassified prokaryotes were found in these locations. This may be explained by the influences of anthropogenic activities altering the biodiversity of marine microbial communities inhabiting the region. Therefore, further investigations, including shotgun metagenomic and transcriptomic approaches, will perhaps provide insights to elucidate such a shift in microbial communities as well as to discover novel bioremediation systems.

### Nucleotide sequence accession numbers.

The DNA sequences of this metagenomic project have been deposited in the NCBI Sequence Read Archive under the accession no. SRP018401 for sample SB and SRP018400 for sample BSM.
